# Determination of Flavonoids, Phenolic Acids, and Xanthines in *Mate* Tea (*Ilex paraguariensis* St.-Hil.)

**DOI:** 10.1155/2013/658596

**Published:** 2013-06-06

**Authors:** Mirza Bojić, Vicente Simon Haas, Darija Šarić, Željan Maleš

**Affiliations:** ^1^Faculty of Pharmacy and Biochemistry, University of Zagreb, A. Kovačića 1, 10000 Zagreb, Croatia; ^2^Universidade Regional Integrada, URI-Farma, Rua Universidade das Missões 464, 98802-470 Santo Ângelo, RS, Brazil

## Abstract

Raw material, different formulations of foods, and dietary supplements of *mate* demands control of the content of bioactive substances for which high performance thin layer chromatography (TLC), described here, presents simple and rapid approach for detections as well as quantification. Using TLC densitometry, the following bioactive compounds were identified and quantified: chlorogenic acid (2.1 mg/g), caffeic acid (1.5 mg/g), rutin (5.2 mg/g), quercetin (2.2 mg/g), and kaempferol (4.5 mg/g). The results obtained with TLC densitometry for caffeine (5.4 mg/g) and theobromine (2.7 mg/g) show no statistical difference to the content of total xanthines (7.6 mg/g) obtained by UV-Vis spectrophotometry. Thus, TLC remains a technique of choice for simple and rapid analysis of great number of samples as well as a primary screening technique in plant analysis.

## 1. Introduction

*Mate* tea (Sp. *yerba mate*, Port. *Erva-mate*) consists of well cut, dried, aerial parts or leaves of *Ilex paraguariensis* St.-Hil. (Aquifoliaceae) shrub. It contains methylxanthines, mainly caffeine, phenolic acids, and saponins and is used as everyday substitution for coffee (psycho stimulant, analeptic) in the south of Brazil, North of Argentina, Oriental Paraguay, and Uruguay. Besides beneficial effects on cardiovascular system, it has significant antioxidant capacity and can be used for the management of weight in obesity [1–3].

Thin layer chromatography (TLC) often represents first-choice technique for samples of plant origin as results can be easily visualized. This technique has been successfully employed for analysis of flavonoids and phenolic acids in wine, propolis, and different medicinal plants [4]. Several studies comparing TLC and HPLC have shown that there is no statistical difference between these methods, and advantage due to lower cost is given to TLC [5, 6]. 

Determination of xanthines as the main constituents is time consuming titration [7]. Although flavonoids present ubiquitous class of compounds, Bastos et al. [8] did not find any of the analyzed flavonols in *mate* tea using HPLC technique. Thus, the goal of this work was to identify and quantify polyphenolic and xanthine components of *mate* densitometrically using HPTLC plates and compare the results obtained with the content of total polyphenols and xanthines based on UV-Vis spectrophotometry. 

## 2. Experimental Section 

### 2.1. Reagents

All standards were purchased from Sigma Aldrich (Germany) and organic solutions from Kemika (Croatia). The sample of *mate* powder (dried aerial parts) was obtained from Santo Ângelo, RS, Brazil.

### 2.2. Standard Solutions

Stock solutions of phenolic acids (chlorogenic, caffeic, ferulic, and *p*-coumaric acid), flavonoids (rutin, quercetin, naringenin, and kaempferol), and xanthines (caffeine, theobromine) were prepared by dissolving standards with methanol (polyphenols) or water (xanthines) to obtain concentration of 1.0 mg/mL. Standard solutions were prepared by dissolution with methanol to 0.1 mg/mL. 

### 2.3. Aqueous Solution of *Yerba Mate*

1.0 g of *erva* was boiled in 100 mL of water for 30 minutes and filtered. 50 mL of this solution was evaporated, and the residues were dissolved in 10 mL of methanol.

### 2.4. Hydrolyzed Solution of *Yerba Mate*

0.5 g of *yerba mate* was refluxed for 30 min. with 20 mL of acetone, 2 mL 25% hydrochloric acid, and 1 mL 0.5% water solution of hexamethylenetetramine. Obtained hydrolyzate was filtered, and crude parts were once again refluxed with acetone for 10 minutes. The filtrates were combined and diluted to 100 mL with acetone. 20 mL of acetone solution was mixed with 20 mL of water and extracted once with 15 mL and 3 times with 10 mL of ethyl acetate. Combined ethylacetate phases were washed with 40 mL of water and filtered through cotton. Filtrate was diluted with ethylacetate to 50 mL. This solution was used for HPTLC analysis and spectrophotometric determination of total flavonoids.

### 2.5. Spectrophotometric Analysis

Total content of flavonoids was determined by Christ-Müller's method, phenolic acids according to European Pharmacopoeia, and xanthines by the method suggested by International Office of Cocoa, Chocolate and Sugar Confectionery [7, 9, 10]. Briefly flavonoids were determined after acid hydrolysis (see Section 2.4.) as liberated aglycones spectrometrically at 425 nm as a complex with AlCl_3_ in a methanol-ethyl acetate-acetic acid medium. 

For the purpose of phenolic acids determination 0.2 g of *mate* powder was refluxed with 190 mL of 50% ethanol on the water bath. Total phenolic acids were determined by measuring the absorbance at 505 nm of the complex formed between phenolic acids and sodium molybdate—sodium nitrite as an equivalent of rosmarinic acid. 

Aqueous solution of *yerba mate* was used for the determination of xanthines. Total xanthines were determined after clarification with lead acetate spectrometrically at 272 nm as an equivalent of theobromine. 

### 2.6. TLC Densitometry

Chloroform : methanol : formic acid in volume ratio 44.1 : 2.5 : 2.15 was used as mobile phase for aglycones, and ethyl acetate : formic acid : acetic acid : water in volume ratio 100 : 11: 11 : 26 was used for glycosides [4, 11]. System used for xanthines development was ethyl acetate, methanol and water in volume ratio 40 : 5.4 : 4 [11]. Merck HPTLC plates (Silica Gel 60 F_254_, 10 × 20 cm) were used as stationary phase. Samples were applied using CAMAG Linomate V semiautomatic sample applicator. After development the HPTLC plates were dried and sprayed for the analysis of polyphenolics with 1% ethanolic solution of AlCl_3_·6H_2_O. Plates were recorded at 254 and 366 nm. Identification and quantification were performed by TLC densitometry using CAMAG TLC Scanner 3 and WinCATS software version 1.3.4.

### 2.7. Statistical Analysis

The results of TLC densitometry and spectrophotometric analysis were compared using Student's *t*-test. Statistical analysis was performed using Microsoft Office Excel 2003.

## 3. Results and Discussion

The total content of flavonoids determined by Christ-Müller's method was 3.0 mg/g (RSD = 0.5%, *n* = 3), phenolic acids determined according to Ph. Eur. method 55.1 mg/g (RSD = 0.6%, *n* = 3), and xanthines by IOCCSC method 7.6 mg/g (RSD = 0.7%, *n* = 3). 

**Figure 1:**
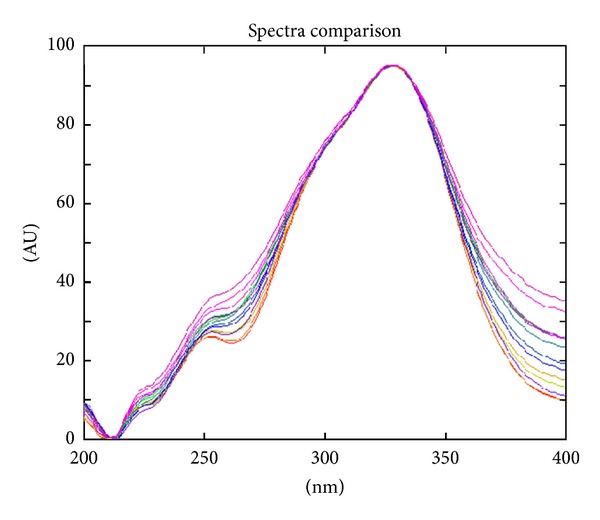
Identification of chlorogenic acid—overlapping spectra of standards and *erva* samples.

Identification of bioactive constituents was based on retention factor, absorption spectra *in situ*, and, if applicable, color of the band after spraying with aluminium chloride. The identification parameters are presented in Table 1 and Figure 1.

**Figure 2:**
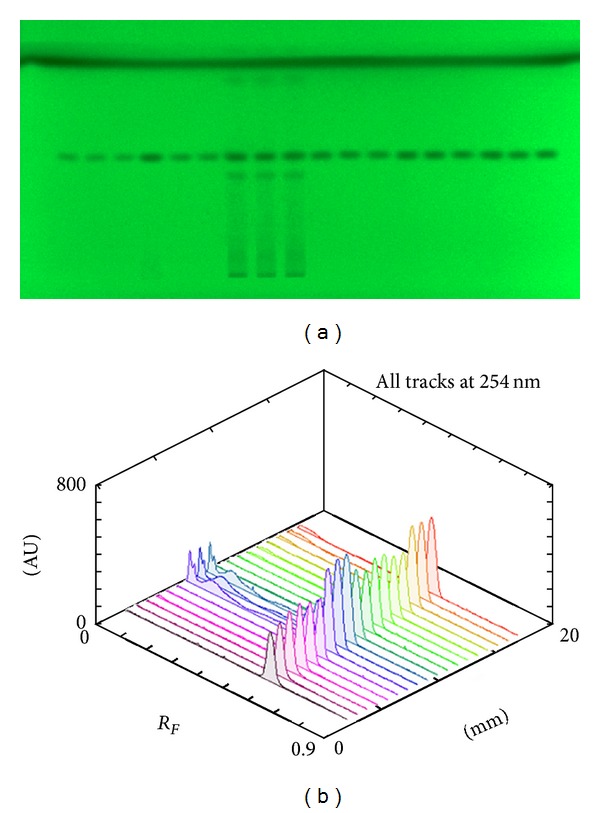
Chromatographic plate (a) and 3D chromatograms (b) of caffeine standard (six tracks from the left and nine from the right) and sample of *mate* (middle part).

Times of chromatogram development were 11.5, 24, and 12 min. for aglycones, glycosides, and xanthines, respectively. Out of eight polyphenolics analyzed rutin, and chlorogenic acid, as well as aglycones quercetin, kaempferol, and caffeic acid were identified and quantified. Both xanthines analyzed, caffeine, and theobromine, were determined (Figure 2). Quantification was based on the area under curve using five bands with different amounts of standard in triplicate. The results of quantification are presented in Table 2.

 Although in the last few years HPLC has been a major method for the analysis of polyphenolics in *yerba mate* and detailed MS analysis was done [12, 13], we have not encountered TLC method in available primary sources. As the TLC is a rather simple technique, we tried to identify and quantify polyphenolic constituents, primarily flavonoids, and phenolic acids of *mate*. Bastos et al. [8] did not detect quercetin, myricetin, and kaempferol in infusion from dried *mate* leaves using HPLC. This could be caused by inappropriate selection of solvents for extraction, inadequate time of extraction, or poor hydrolysis of glycosides as aglycones were to be determined. As it can be noticed from the result in Table 2 most commonly employed acid hydrolysis used for the determination of total flavonoids in aglycone form (Christ-Müller's method) did not provide complete degradation of glycoside rutin. 

Although advanced techniques for the separation and quantification of individual polyphenols including LC-MS [13, 14] are available, we used simple TLC method with HPTLC plates to determine quercetin and kaempferol in *yerba mate* in the amounts of 2.2 mg/g and 4.5 mg/g, respectively.

The results of TLC analysis concurred with the total content of methylxanthines: 5.4 mg/g for caffeine and 2.7 mg/g for theobromine, while total xanthines were 7.6 mg/g (*t* = 0.16,   *α* = 0.01). These results suggest that thin layer chromatography with densitometry could be used for both, identification as well as determination of caffeine and theobromine in *yerba mate* having in mind that the most common method only for determination is time-consuming titration [8].

## 4. Conclusion

Based on the obtained results, it can be concluded that TLC analysis is an appropriate technique for the analysis of individual polyphenols as well as xanthines. Content of individual methylxanthines corresponds to the spectrophotometric determined total xanthines. 

As thin layer chromatography is readily available technique, it could be easily employed for the analysis of polyphenols and xanthines in raw material, different formulations of foods, and dietary supplements of *mate*. 

## Figures and Tables

**Table 1:** Identification parameters of polyphenolics and xanthines: retention factor, color of band under 366 nm, and spectra after spraying with aluminium chloride (not applicable for caffeine and theobromine).

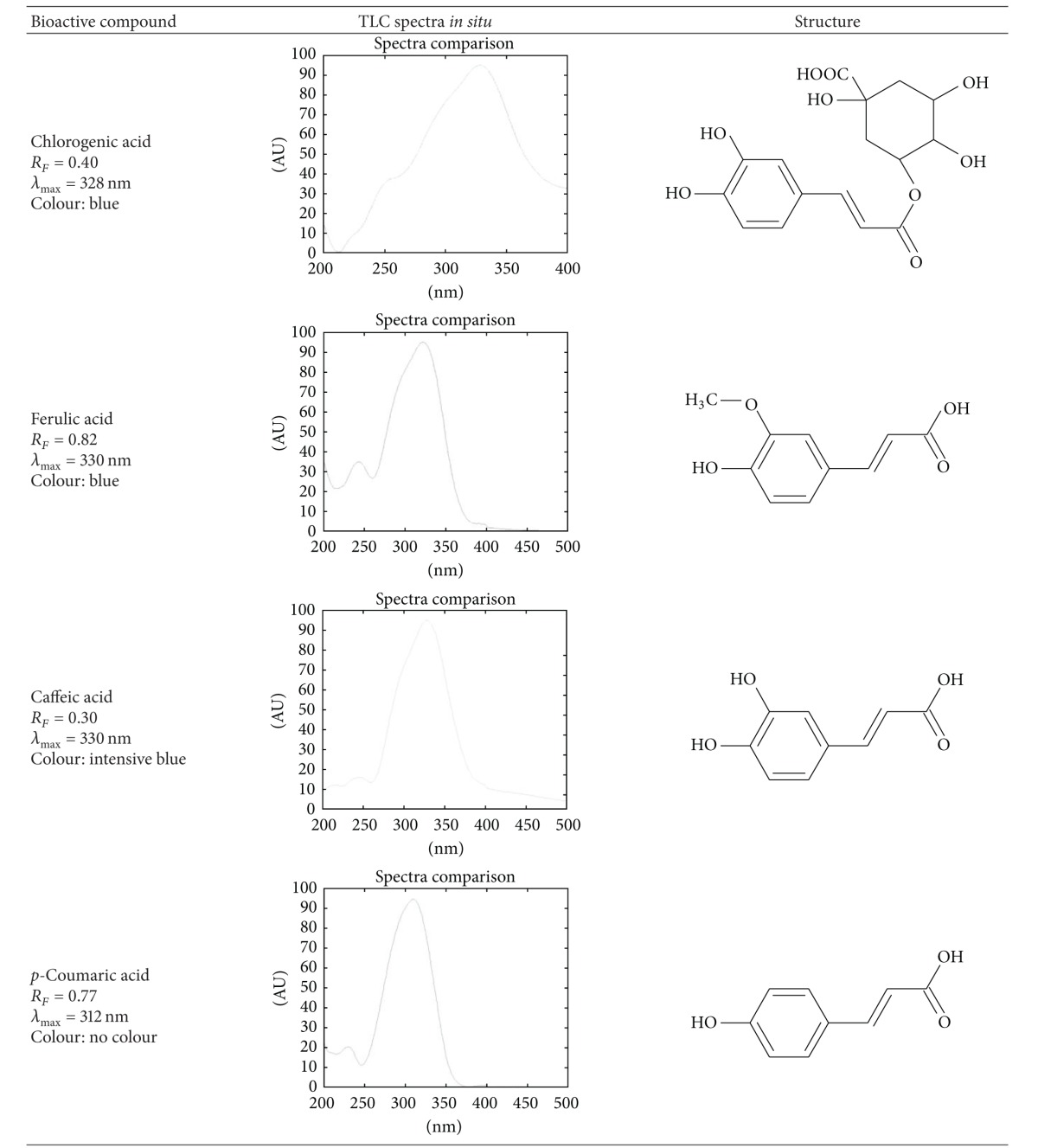 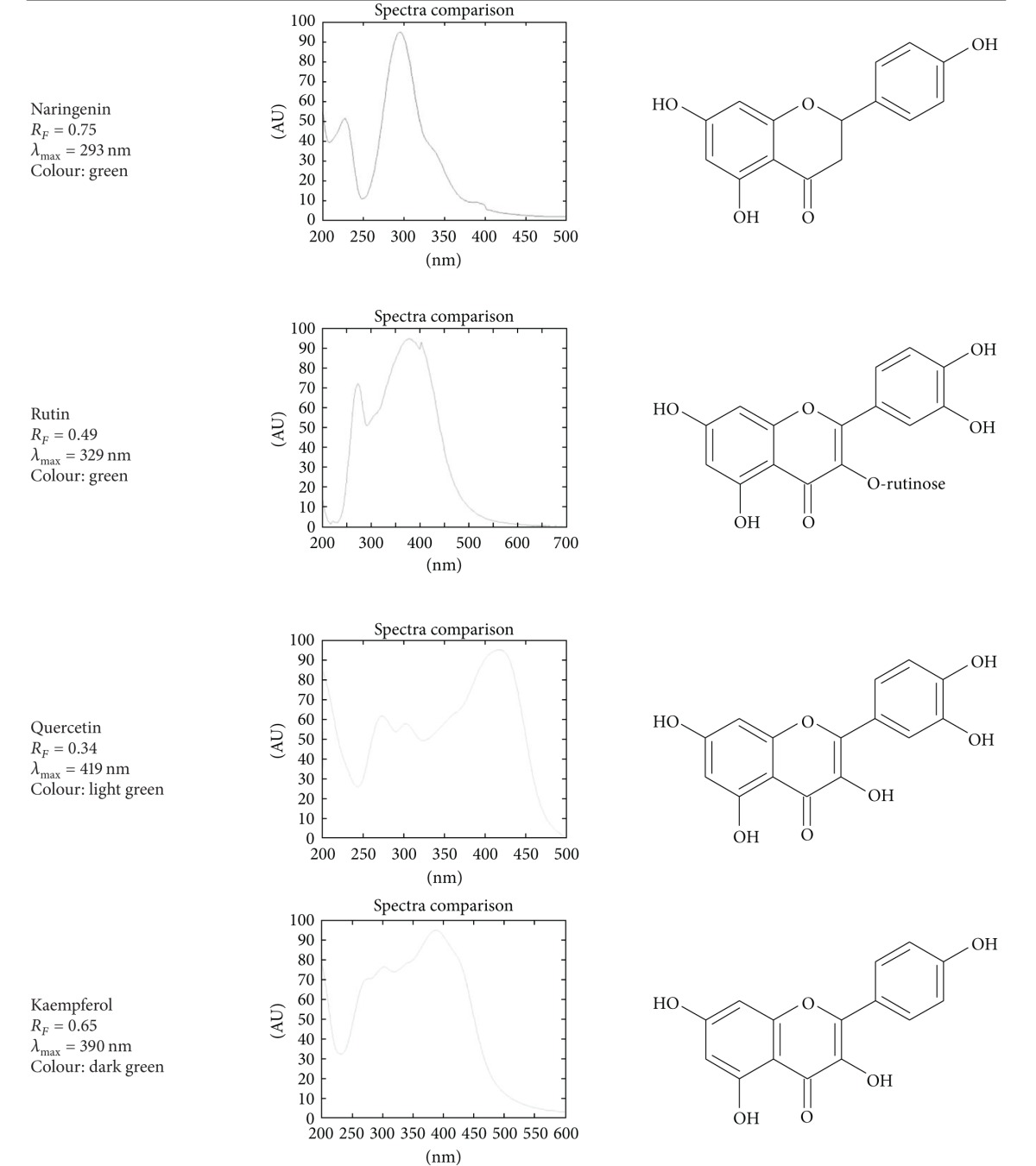 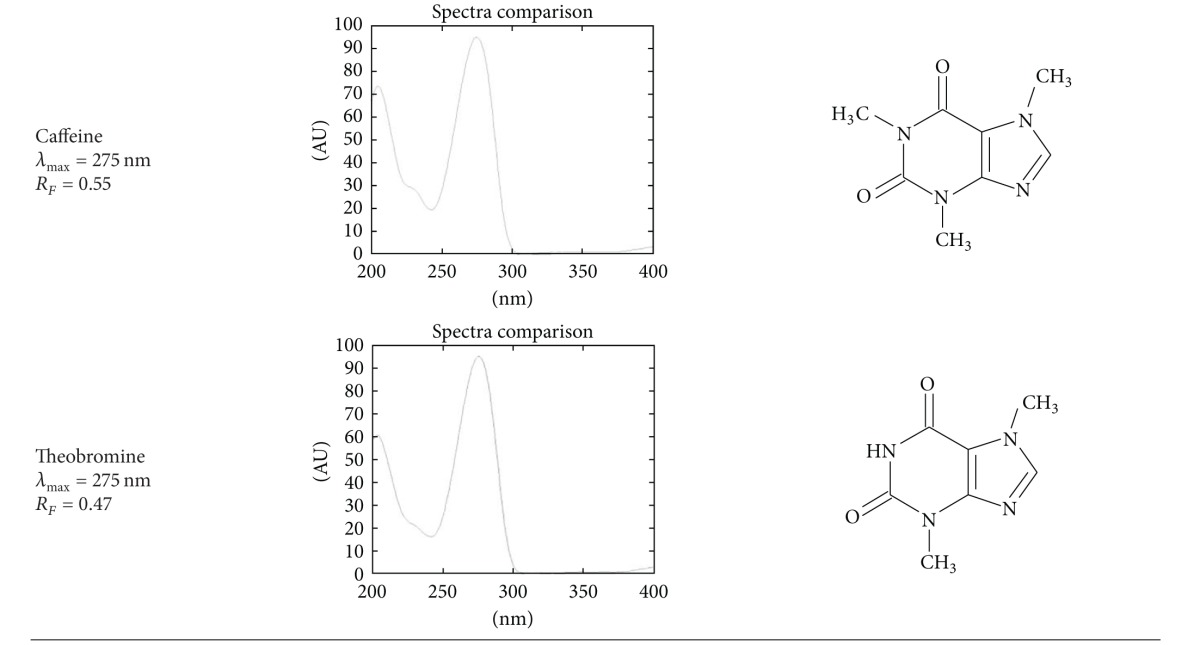

**Table 2:** Results of quantification of individual flavonoids, phenolic acids, and xanthines.

Bioactive compound	Calibration curve	Fraction of flavonoids^1^	Fraction of phenolic acids^2^	Aqueous solution
		(mg/g)	(mg/g)	(mg/g)
Chlorogenic acid	*A* = 3168.1*m* + 204.2 (*r* ^2^ = 0.9865)	2.1	21.6	3.0
Caffeic acid	*A* = 3313.2*m* − 475.6 (*r* ^2^ = 0.9955)	1.5	nd	nd
Rutin	*A* = 6223.4*m* − 649.8 (*r* ^2^ = 0.9833)	5.2	6.8	2.3
Quercetin	*A* = 6902.1*m* + 723.7 (*r* ^2^ = 0.9788)	2.2	nd	nd
Kaempferol	*A* = 9349.3*m* − 732.7 (*r* ^2^ = 0.9681)	4.5	nd	nd
Caffeine	*A* = 2328.8*m* + 6439.2 (*r* ^2^ = 0.9807)	na	na	5.4
Theobromine	*A* = 3405.5*m* + 1535.9 (*r* ^2^ = 0.9667)	na	na	2.7

^1^Hydrolyzed fraction of flavonoids, ^2^nonhydrolyzed fraction of phenolic acids used for the spectrophotometric analysis according to the European pharmacopoeia, and nd: not detected, na: not applicable.
